# Enteropathogenic *Escherichia coli* (EPEC) Recruitment of PAR Polarity Protein Atypical PKCζ to Pedestals and Cell–Cell Contacts Precedes Disruption of Tight Junctions in Intestinal Epithelial Cells

**DOI:** 10.3390/ijms21020527

**Published:** 2020-01-14

**Authors:** Rocio Tapia, Sarah E. Kralicek, Gail A. Hecht

**Affiliations:** 1 Department of Medicine, Division of Gastroenterology and Nutrition, Loyola University Chicago, Maywood, IL 60153, USA; rtpastrana@luc.edu (R.T.); sknopf@luc.edu (S.E.K.); 2 Department of Microbiology and Immunology, Loyola University Chicago, Maywood, IL 60153, USA; 3 Edward Hines Jr. VA Hospital, Hines, IL 60141, USA

**Keywords:** enteropathogenic *E. coli* (EPEC), tight junctions (TJ), polarity, atypical aPKCζ, transepithelial electrical resistance (TER), sorting nexin 9 (SNX9), EspF

## Abstract

Enteropathogenic *Escherichia coli* (EPEC) uses a type three secretion system to inject effector proteins into host intestinal epithelial cells, causing diarrhea. EPEC induces the formation of pedestals underlying attached bacteria, disrupts tight junction (TJ) structure and function, and alters apico-basal polarity by redistributing the polarity proteins Crb3 and Pals1, although the mechanisms are unknown. Here we investigate the temporal relationship of PAR polarity complex and TJ disruption following EPEC infection. EPEC recruits active aPKCζ, a PAR polarity protein, to actin within pedestals and at the plasma membrane prior to disrupting TJ. The EPEC effector EspF binds the endocytic protein sorting nexin 9 (SNX9). This interaction impacts actin pedestal organization, recruitment of active aPKCζ to actin at cell–cell borders, endocytosis of JAM-A S285 and occludin, and TJ barrier function. Collectively, data presented herein support the hypothesis that EPEC-induced perturbation of TJ is a downstream effect of disruption of the PAR complex and that EspF binding to SNX9 contributes to this phenotype. aPKCζ phosphorylates polarity and TJ proteins and participates in actin dynamics. Therefore, the early recruitment of aPKCζ to EPEC pedestals and increased interaction with actin at the membrane may destabilize polarity complexes ultimately resulting in perturbation of TJ.

## 1. Introduction

Enteropathogenic *Escherichia coli* (EPEC) delivers bacterial effector proteins into host intestinal epithelial cells (IECs) through a type III secretion system (TTSS), inducing actin pedestal formation, attaching and effacing lesions, and physiological changes in IECs that contribute to diarrhea [1]. EPEC alters the architecture and barrier function of tight junctions (TJ) [2,3] although the mechanisms are not well understood.

TJ are localized at the most apical region of the lateral membrane and constitute a paracellular diffusion barrier modulating the flow of ions and solutes. These structures consist of integral membrane proteins (claudin family, occludin, tricellulin, MarvelD3, and JAM-A) that interact with adhesion molecules of adjacent cells and with intracellular domains that associate with cytoplasmic adaptor proteins (MAGUK family, cingulin, paracingulin, MAGI-1-3, and MUPP-1) [4,5]. TJ also constitute a fence contributing to the maintenance of apico-basal polarity by restricting the intermixing of apical and lateral plasma membrane components. Three main protein complexes control epithelial polarity, Crumbs (Crb3/Pals1/Patj), PAR (Par3/Par6/aPKCζ/Cdc42), and Scribble (Scrib/Lgl/Dlg). Apico-basal polarity contributes to cell morphology, directional vesicle transportation, ion and solute transport, and specific localization of proteins and lipids to different membrane domains [6,7]. 

The interdependence between apico-basal polarity complexes and TJ is well established. Alterations in Crb3 expression or reduced expression of Patj/Pals1 impair apical polarity and TJ development [8,9,10,11,12]. Inhibition of aPKCζ activity, impaired phosphorylation of Par3, as well as deletion of the aPKCζ binding domain of Par6, delays TJ assembly [13,14,15,16]. aPKC activity also maintains TJ integrity and membrane localization of occludin and ZO-1 [17]. Downregulation of Scrib or Dlg compromises TJ establishment [18,19,20]. In contrast, increased expression of Scrib in MCF10A cells promotes the formation of functional TJ [21]. These data demonstrate that polarity complexes are crucial to TJ assembly, maintenance, and function.

EPEC effectors perturb TJ structure and function and alter apico-basal polarity of IECs. EspF perturbs barrier function in vivo and in vitro by redistributing TJ proteins from the cell–cell contacts, decreasing transepithelial electrical resistance (TER), and increasing paracellular permeability [2,22,23,24]. Map increases permeability to charged and non-charged molecules, indicating a failure in gate function [24,25]. NleA mislocalizes occludin and ZO-1 from the cell–cell contacts leading to barrier dysfunction [26]. EspG also contributes to leaky barrier, perturbs microtubule networks, and induces cytoplasmic accumulation of occludin and delays TJ recovery [27,28,29].

EPEC infection causes progressive redistribution of the basolateral proteins, β1-integrin and Na^+^/K^+^ ATPase, to the apical compartment and the mislocalization of TJ proteins, occludin, claudin-1, and ZO-1 from cell–cell contacts to the lateral membrane and cytoplasm [22,23,26,30,31,32], suggesting that cell polarity is altered. We recently reported that EPEC drives Crb3 and Pals1 away from the apical membrane, and cell–cell contacts into the cytoplasm of IECs and EspF is crucial for this phenotype [32]. EspF is a multifunctional molecule that interacts with several host proteins including actin, profilin, Arp2, N-WASP, SNX9, Abcf2, cytokeratin 18, 14-3-3, WIPF1, SNX18, and SNX33 [33,34,35,36,37,38]. EspF interacts with the SH3 domain of sorting nexin 9 (SNX9) through its RxAPxxP motif [33,35]. The interaction of EspF with SNX9 promotes the formation of elongated plasma membrane tubules, as well as the internalization of EPEC into IECs [39]. EspF/SNX9 complex is required for impairment of both cell polarity and altered TJ structure and function [32,33,40].

Despite extensive investigation into the mechanisms by which EPEC effectors directly perturb TJ, no such evidence has been reported. In view of the interdependence of polarity and TJ complexes, we hypothesized that EPEC-induced disruption of intestinal epithelial cell TJ structure and function stems from the initial targeting of polarity complexes. This study examines the effect of EPEC on the PAR complex with particular focus on aPKCζ, which phosphorylates several targets crucial for the establishment and maintenance of apico–basal polarity and TJ function. The data presented herein support the notion that EPEC-induced perturbation of TJ is a downstream consequence of EspF-induced disruption of the PAR polarity complex, particularly the recruitment of aPKCζ to actin-rich pedestals, and its increased co-localization with actin at the membrane.

## 2. Results

### 2.1. EPEC Disrupts PAR Polarity Complexes In Vivo and In Vitro

**Figure 1:**
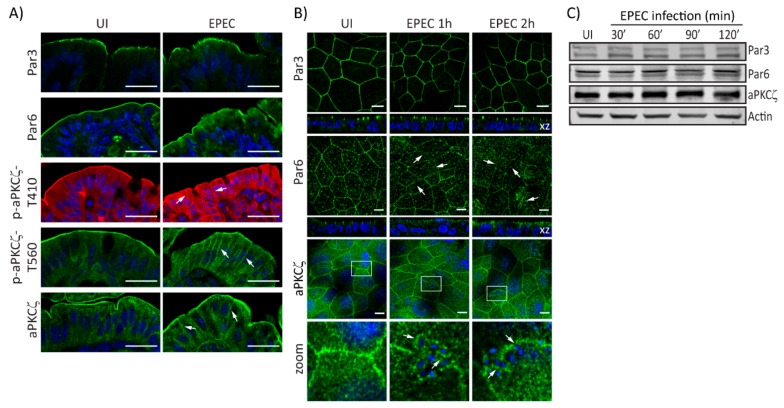
EPEC alters PAR complex localization. (**A**) Immunofluorescence microscopy of murine colonic epithelial tissue 3 days post-infection with EPEC or not (uninfected—UI). Par3 localization is unchanged. Par6 accumulates within the cytoplasm. Phosphorylated aPKCζ–T410, aPKCζ–T560, and total aPKCζ mislocalize from the apical membrane and accumulate within the cytoplasm and lateral membrane (arrows). Scale bar: 40 µm. (**B** and **C**) SKCO-15 monolayers were infected, or not (UI), with EPEC at indicated times, then the localization and protein levels of PAR complex proteins were assessed by immunofluorescence and western blot, respectively. (**B**) Par3 localization is unaltered by EPEC infection, and Par6 redistributes from cell–cell contacts to the cytoplasm (arrows). aPKCζ localizes under attached bacteria (arrows, inset zoom). Hoechst was used to stain host and bacteria nuclei (blue). Scale bar: 10 µm. (**C**) EPEC does not change the expression levels of PAR complex proteins; β-actin was used as a loading control.

EPEC alters the localization of Crb complex proteins resulting in perturbed cell polarity [32]. Here, we investigate the effect of EPEC on PAR polarity components. EPEC does not change the localization of Par3 in murine colonocytes as compared with uninfected (UI) tissues (Figure 1A). In contrast, colonocytes of EPEC-infected mice show that Par6 is redistributed from the apical membrane to the cytoplasm (Figure 1A). Similarly, phosphorylated aPKCζ–T410, aPKCζ–T560, and total aPKCζ in the apical membrane of colonocytes of EPEC-infected mice appear diffuse and shift from the apical to the lateral membrane and into the cytoplasm compared to its restricted localization to the apical membrane in UI cells (Figure 1A). To begin to explore the mechanisms involved in PAR complex perturbation caused by EPEC, in vitro models were used. SKCO-15 monolayers were infected for 1-2 h and the localization and protein levels of PAR complex proteins were examined. In accordance with in vivo data, Par3 localization in cultured IECs was unchanged by EPEC infection (Figure 1B). In contrast, EPEC displaced Par6 from cell–cell contacts into the cytoplasm (Figure 1B). Interestingly, aPKCζ displayed a ringlet pattern around attached bacteria (Figure 1B, zoom). Expression levels of PAR complex proteins are not altered by EPEC infection (Figure 1C). These data demonstrate that EPEC alters the distribution of the PAR complex proteins, Par6 and aPKCζ without changing their expression levels.

### 2.2. EspF, Via Its SNX9-Binding Domain, Impacts the Structural Organization of aPKCζ and F-actin at Pedestals

**Figure 2:**
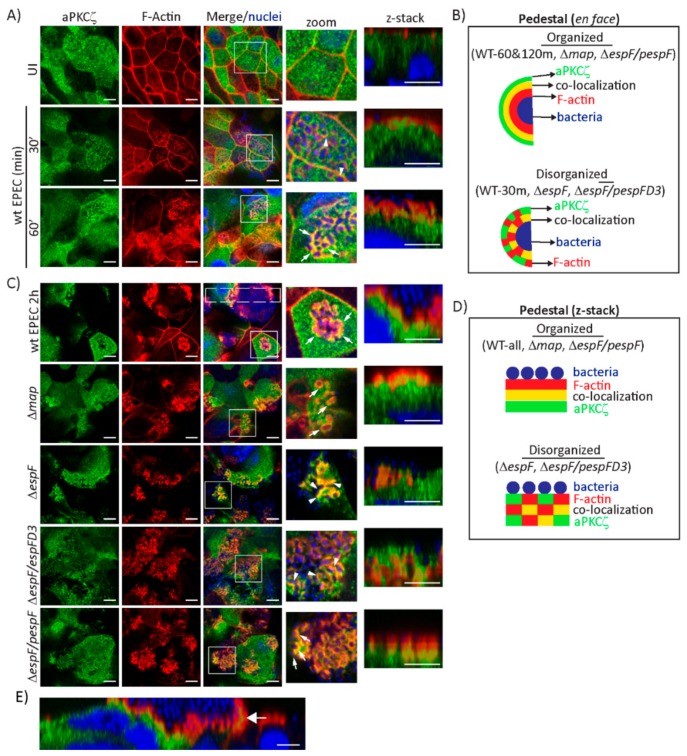
EspF and its SNX9-binding domain are crucial for the structural organization of F-actin and aPKCζ within EPEC pedestals. SKCO-15 monolayers were infected with wt EPEC, *Δmap, ΔespF*, *ΔespF/pespF*D3, or *ΔespF/pespF* to assess F-actin and aPKCζ co-localization. (**A**) F-actin and aPKCζ are recruited to and co-localize around attached bacteria at 30 and 60 min post-infection with wt EPEC. Zoom and z-stack images at 60 min display EPEC pedestals with attached bacteria surrounded by F-actin, an interface of co-localized F-actin and aPKCζ, then aPKCζ alone. (**B**) Schematic of F-actin (red), aPKCζ (green), and co-localizing interface (yellow) organization around bacteria after infection with wt EPEC and mutant strains. (**C**) aPKCζ recruitment to pedestals is maintained at 2 h post-infection with wt EPEC. Zoom and z-stacks reveal the robust organization of F-actin and aPKCζ co-localization. Deletion of *map* (*Δmap*) does not alter aPKCζ recruitment to pedestals. Infection with *ΔespF* and *ΔespF/pespF*D3 causes disorganization of co-localized F-actin and aPKCζ within pedestals. Complementation of *espF* (*ΔespF/pespF*) restores the organized phenotype. Squares correspond to zoom and z-stack areas in all except wt EPEC, in which a large rectangle corresponds to z-stack image. Arrows and arrowheads indicate organized and disorganized pedestals, respectively. (**D**) Schematic representation of pedestals showing the organized and disorganized localization of F-actin (red), aPKCζ (green), and co-localizing interface (yellow) after infection with wt EPEC and mutant strains. (**E**) aPKCζ also co-localizes with F-actin in filopodia (arrow) 2 h post-infection with wt EPEC. Scale bars: 10 µm (en face); 5 µm (z-stack).

EPEC induces the translocation of aPKCζ from the cytoplasm to the plasma membrane [41,42]. aPKCζ localization under attached EPEC in SKCO-15 cells suggests it is recruited to actin-rich pedestals. Indeed, aPKCζ co-localizes with filamentous actin (F-actin) around attached bacteria as early as 30 min and becomes more evident at 60 min post-infection (Figure 2A). The pattern around attached bacteria is highly organized in which actin surrounds bacteria, then a ring of aPKCζ and F-actin co-localization, and finally aPKCζ alone as seen in Figure 2A zoom, and depicted in the schematic in Figure 2B. Confocal microscopy confirms pedestal organization at 60 min post-infection consisting of attached bacteria atop actin, an interface of actin and aPKCζ co-localization, and then a region of aPKCζ alone (Figure 2A z-stack and Figure 2B). This structural organization is maintained at 2 h post-infection (Figure 2C zoom and z-stack, Figure 2B,D). Interestingly, aPKCζ is also seen within large filopodia induced by attached EPEC co-localizing with actin (Figure 2E).

We previously demonstrated that the effectors Map and EspF, through its interaction with SNX9, disrupt the Crb polarity complex [32]. Therefore, we investigated the contribution of Map and EspF to the recruitment of aPKCζ to pedestals. aPKCζ recruitment to EPEC pedestals is not diminished by deletion of *map* (*Δmap*) or *espF* (*ΔespF*), or by mutation of the SNX9-binding domain of EspF (*ΔespF/pespF*D3) (Figure 2C, green channel). However, the localization of F-actin and aPKCζ within pedestals is disorganized when infected with *ΔespF* and *ΔespF/pespF*D3, but not *Δmap*, compared to infection with wild-type (wt) EPEC, seen both en face and within z-stack images (Figure 2B–D). Complementation of *espF* (*ΔespF/pespF*) restores the organization of actin and aPKCζ within pedestals (Figure 2C, zoom and z-stack, Figure 2B,D). 

Interestingly, infection of T84 monolayers with *ΔespF/pespF*D3 produces aPKCζ aggregates under attached bacteria in actin pedestals similar to wt EPEC and *ΔespF/pespF*, while only infection with *ΔespF* induces less co-localization of aPKCζ and actin (Supplemental Figure S1A,B). These data indicate that aPKCζ recruitment to pedestals is not dependent on EspF, however, the SNX9-binding domain of EspF is required for the structural organization of actin and aPKCζ within pedestals in a cell-specific manner.

### 2.3. EspF and Its SNX9-Binding Domain Contribute to the Recruitment of Phosphorylated aPKCζ–T560 to Pedestals without Altering Kinase Activity

**Figure 3:**
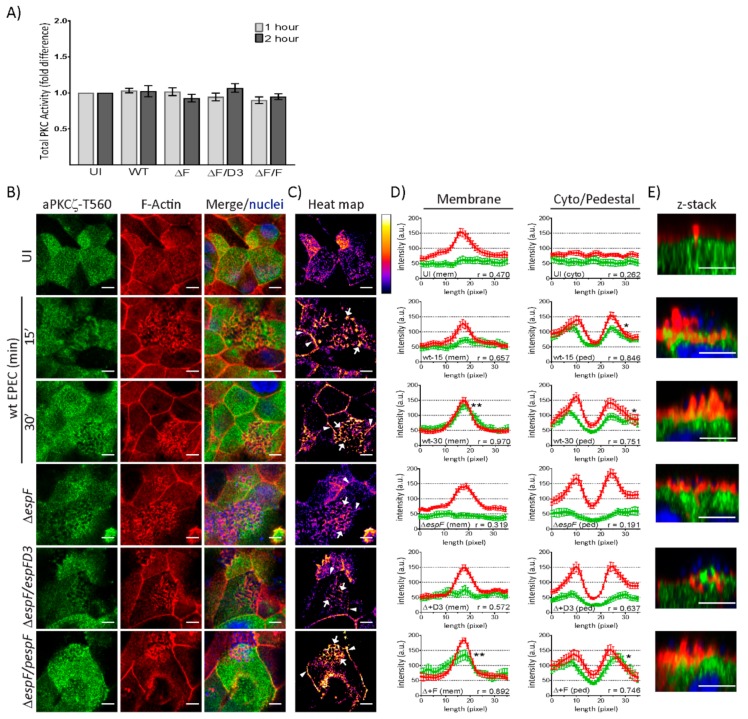
EspF and its SNX9-binding domain contribute to the co-localization of phosphorylated aPKCζ–T560 and F-actin at the plasma membrane and within pedestals. SKCO-15 monolayers plated on Transwells were infected with wt EPEC or EspF mutant strains, then PKC kinase activity, and F-actin and p-aPKCζ-T560 co-localization were assessed. (**A**) Fold change in PKC activity does not change at 1 or 2 h post-infection with EPEC (WT), *ΔespF* (ΔF), *ΔespF* complemented with mutant *espF* (ΔF/D3) or wt *espF* (ΔF/F) compared to UI monolayers. (**B**–**E**) p-aPKCζ–T560 is recruited to and co-localizes with F-actin at the cell–cell contacts and within pedestals 15 and 30 min post-infection with wt EPEC. p-aPKCζ–T560 recruitment is reduced following infection with *ΔespF* or *ΔespF/pespF*D3. Complementation of *espF* (*ΔespF/pespF*) restores F-actin and p-aPKCζ–T560 co-localization levels similar to wt. (**C**) Heat map generated from merged images in B indicating the intensity of F-actin and p-aPKCζ–T560 co-localization at the plasma membrane (arrowheads) and pedestals (arrows). (**D**) Immunofluorescence (IF) quantification of images represented in B. IF intensity in arbitrary units (a.u.) plotted against length (pixel) of p-aPKCζ–T560 (green) and F-actin (red) at the membrane and within the cell. Graphs represent data from >15 regions of interest from three biological replicates. Average peak intensity for p-aPKCζ–T560 reveals significantly higher levels at the membrane with wt and *ΔespF/pespF* at 30 min post-infection and significantly higher levels within pedestals with wt-15, wt-30, and *ΔespF/pespF* 30 min post-infection compared to uninfected controls; one-way ANOVA, * *p* < 0.01 and ** *p* < 0.001. r = Pearson correlation coefficient indicating an association between F-actin and p-aPKCζ–T560 localization at the membrane and within pedestals in an EspF and SNX9-binding domain dependent manner. (**E**) Z-stack images reveal F-actin and p-aPKCζ–T560 co-localization within pedestals. Scale bars: 10 µm (en face); 5 µm (z-stack).

To determine if EPEC infection alters kinase activity, total PKC activity was measured in T84 and SKCO-15 monolayers following infection with wt EPEC or EspF mutant strains for 1–2 h. In agreement with our previous observations, a significant increase in the kinase activity of total PKC in T84 cells is observed following infection with EPEC and EspF mutant strains (Supplementary Figure S1C) [41,42]. Interestingly, EPEC failed to alter the kinase activity of total PKC in SKCO-15 monolayers (Figure 3A). aPKCζ autophosphorylation at Thr560 (p-aPKCζ–T560) activates kinase activity [43,44]. We questioned whether active aPKCζ is recruited to pedestals. Figure 3B shows the co-localization of p-aPKCζ-T560 with F-actin in EPEC at the cell–cell borders and pedestals. Heat maps were generated by merging the green and red channels depicted in Figure 3B in order to visually quantitate the co-localization of p-aPKCζ–T560 with F-actin; white indicates the most abundant co-localization and blue-black the least to none (Figure 3C). To more specifically quantify F-actin and p-aPKCζ–T560 co-localization, immunofluorescence intensity was measured in regions of interest within cell–cell contacts, the cytoplasm, or pedestals (Figure 3D). In uninfected cells, there is no significant association of F-actin and p-aPKCζ–T560 at cell membranes or within the cytoplasm (Figure 3D), despite their apparent co-localization (Figure 3B,C). However, co-localization within the membrane intensifies as early as 15 min post-infection with wt EPEC (Figure 3B,C) and significant correlation between F-actin and p-aPKCζ–T560 is present at 30 min post-infection (Figure 3D). Interestingly, at both 15 and 30 min post-infection with wt EPEC, there is significant association between p-aPKCζ–T560 and F-actin within pedestals (Figure 3B–D) and confocal microscopy confirms this co-localization (Figure 3E). Interestingly, both *ΔespF* and *ΔespF/pespF*D3 diminish p-aPKCζ–T560 and F-actin co-localization at both the plasma membrane and within pedestals 30 min post-infection (Figure 3B–E). Complementation of *ΔespF* with wt EspF (*ΔespF/pespF*) restores the interaction of F-actin and p-aPKCζ–T560 at cell–cell borders and pedestals (Figure 3B–E). Together, these results suggest that although PKC activity is unchanged by EPEC infection in SKCO-15 cells, active aPKCζ localization within the cell is altered and is dependent on EspF and its SNX9-binding domain for the recruitment of p-aPKCζ–T560 to actin at cell–cell borders and EPEC-induced pedestals.

### 2.4. SNX9-Binding Domain of EspF Is Essential to Disrupt TJ Structure and Function in SKCO-15 Monolayers

**Figure 4:**
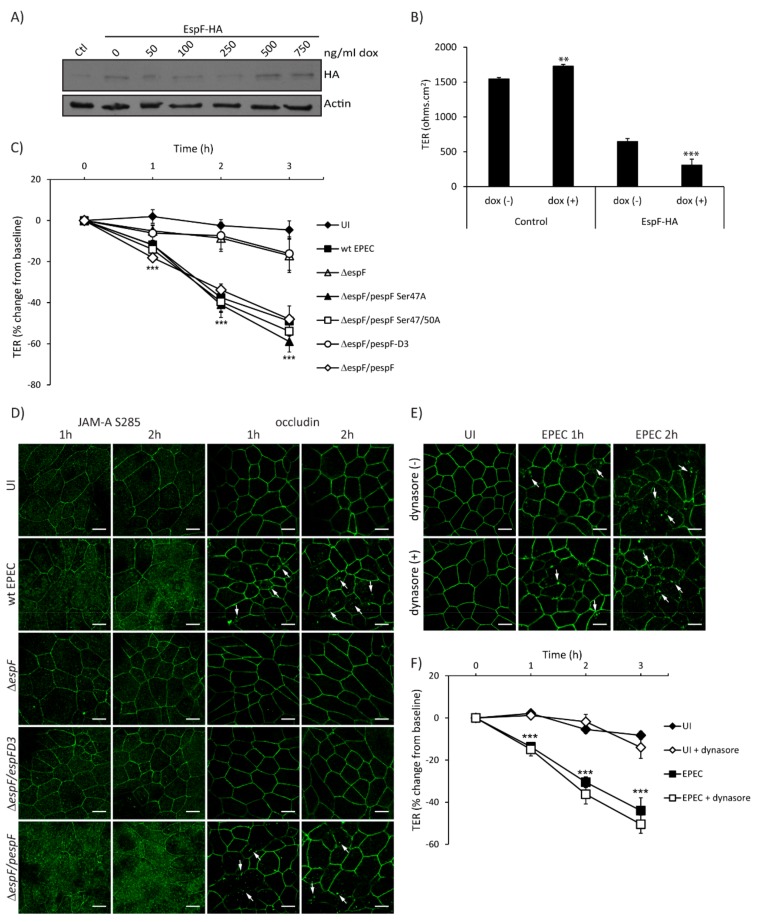
The SNX9 binding domain of EspF is crucial for disruption of TJ structure and function. (**A** and **B**) To generate Tet–On cells, EspF–HA was cloned into the doxycycline-inducible p–RetroX–Tight–Pur vector and transfected or not (control – Ctl) into wild-type SKCO-15 cells. Tet–On SKCO-15 cells were plated in absence of doxycycline (dox). (**A**) EspF–HA expression was induced with increasing concentrations of dox for three days. Cells were processed for western blot and detection of HA was performed. (**B**) Tet–On SKCO-15 cells were cultured on Transwells for 1 week (-dox) then expression of pRetroX–Tight–Pur–EspF-HA was induced (+dox) for three days. Basal expression of EspF–HA induces a significant reduction in TER (-dox) as compared to controls. Induction of EspF–HA (+dox) significantly reduces TER even further. (**C** and **D**) SKCO-15 monolayers were infected with wt EPEC, *ΔespF*, or *ΔespF* complemented with wt *espF* or site-directed *espF* mutants (*ΔespF/pespF*, *ΔespF/pespF*D3, *ΔespF/pSer47A*, or *ΔespF/pSer47/50A*). TER and localization of JAM-A S285 and occludin were determined. (**C**) Infection with *ΔespF/pespF, ΔespF/pSer47A* and *ΔespF/pSer47/50A* mutant strains reduce TER to levels similar to those induced by wt EPEC infection. Infection with *ΔespF* or *ΔespF/pespF*D3 significantly blunt the decrease in TER compared to wt EPEC. ***p < 0.001. (**D**) Infection with wt EPEC and *ΔespF/pespF* redistributes JAM-A S285 and occludin from intercellular junctions to the cytoplasm. Infection with *ΔespF* or *ΔespF/pespFD3* does not alter the localization of JAM-A S285 and occludin. Scale bar, 10 µm. (**E** and **F**) SKCO-15 monolayers were treated with or without dynasore 1 h prior to EPEC infection, then occludin localization and TER were assessed. (**E**) Blocking dynamin-dependent endocytosis with dynasore fails to prevent occludin internalization induced by wt EPEC infection. Arrows indicate the endocytosis of occludin triggered by EPEC infection. Scale bar, 10 µm. (**F**) Dynasore does not prevent the drop in TER induced by EPEC. TER reported as percent change from baseline. *p < 0.01, ***p < 0.001.

EPEC effectors contribute to loss of intestinal epithelial TJ structure and function, and EspF is largely responsible for this phenotype [2]. To determine the individual contribution of EspF to barrier disruption, TER was measured in SKCO-15 monolayers harboring pRetroX–Tight–Pur–EspF-HA with and without inducible expression. EspF-HA expression levels were determined by western blot of control and Tet–On cells incubated with increasing amounts of doxycycline (dox) (Figure 4A). The basal expression of EspF–HA is observed in the absence of dox compared to control cells and increases at 500 ng/mL of dox (Figure 4A). Control and EspF–HA cells were grown in Transwells with and without dox (500 ng/mL) for three days, then TER was determined. Interestingly, uninduced (-dox) cells transfected with EspF–HA show a significant reduction in TER values as compared to control monolayers (647 ± 44 ohms.cm^2^ vs. 1545 ± 22 ohms.cm^2^) (Figure 4B), likely due to basal expression of EspF (Figure 4A) as previously reported [32]. Induced expression of EspF–HA (+dox) significantly decreases TER compared to uninduced (-dox) (308 ± 86 ohms.cm^2^ vs. 647 ± 44 ohms.cm^2^) (Figure 4B) indicating that EspF alone significantly reduces TER in SKCO-15 monolayers. 

To assess in detail the EspF motifs involved in perturbation of TJ barrier function, SKCO-15 monolayers were infected with wt EPEC, *ΔespF*, *ΔespF/pespF,* or *ΔespF* complemented to express specific site-directed EspF mutations (*ΔespF/pespF*D3, *ΔespF/pSer47A*, and *ΔespF/pSer47/50A*). In addition to binding SNX9, EspF binds to 14-3-3 family proteins [36]. EPEC strains expressing EspF harboring 14-3-3ζ binding motif mutations (*ΔespF/pSer47A* and *ΔespF/pSer47/50A)* were used as controls because of their inability to perturb the Crb polarity complex [32]. Infection with *ΔespF/pSer47A* and *ΔespF/pSer47/50A* perturbed barrier function to a similar degree as wt EPEC (–41.0 ± 6.0% and –40.0 ± 5.0% vs. –38.0 ± 7.0%, change from baseline, respectively) (Figure 4C). In contrast, *ΔespF* and *ΔespF*/*pespF*D3 mutants induced minimal change in TER at 2 h compared to wt EPEC (–9.0 ± 7.0% and –7.0 ± 6.0% vs. –38.0 ± 7.0%, change from baseline, respectively) (Figure 4C). As reported previously in T84 cells [33], *ΔespF/pespF*D3 decreased TER to the same extent as wt EPEC (–35.7 ± 5.8% vs. –32.3 ± 5.1%, change from baseline) (Supplemental Figure S1D). Infection with *ΔespF* attenuates the decrease in resistance (–18.3 ± 5.1%, change from baseline) and complementation of *espF* (*ΔespF/pespF*) restores this phenotype (–38.6 ± 7.8%, change from baseline) (Supplemental Figure S1D), indicating the cell-specific responses to the EspF SNX9-binding domain mutant. 

We next questioned whether EspF and the SNX9-binding domain play a role in the maintenance of TJ structure and function. We evaluated the distribution of phosphorylated JAM-A at Ser285 and occludin, which are both involved in the assembly and maintenance of TJ. Interestingly, *ΔespF/pespF*D3 ablates the redistribution of JAM-A S285 and occludin from the membrane to the cytoplasm caused by wt EPEC in a similar manner to those cells infected with *ΔespF* (Figure 4D). We previously demonstrated that the EspF/SNX9 interaction is crucial for the endocytosis of Crb3 in a clathrin-dependent manner [32]. We therefore investigated whether the redistribution of occludin caused by EPEC is mediated by a similar endocytic pathway. SKCO-15 monolayers were incubated, or not, with dynasore, a dynamin inhibitor that plays an important role in clathrin-dependent endocytosis, and infected, or not, with EPEC for 1–2 h. Occludin localization and TER measurements were followed. Treatment with dynasore did not prevent the endocytosis of occludin (Figure 4E) or the decrease in TER caused by EPEC at 1–2 h post-infection (–15.0 ± 3.0% vs. –14.0 ± 2.0%; –36.0 ± 5.0% vs. –30.0 ± 2.0%, with and without dynasore at 1 and 2 h post-infection, respectively) (Figure 4F). These results indicate that occludin endocytosis and barrier dysfunction mediated by EPEC occurs via a dynamin-independent pathway.

### 2.5. Temporal Disruption of TJ Proteins Mediated by EPEC Infection

**Figure 5:**
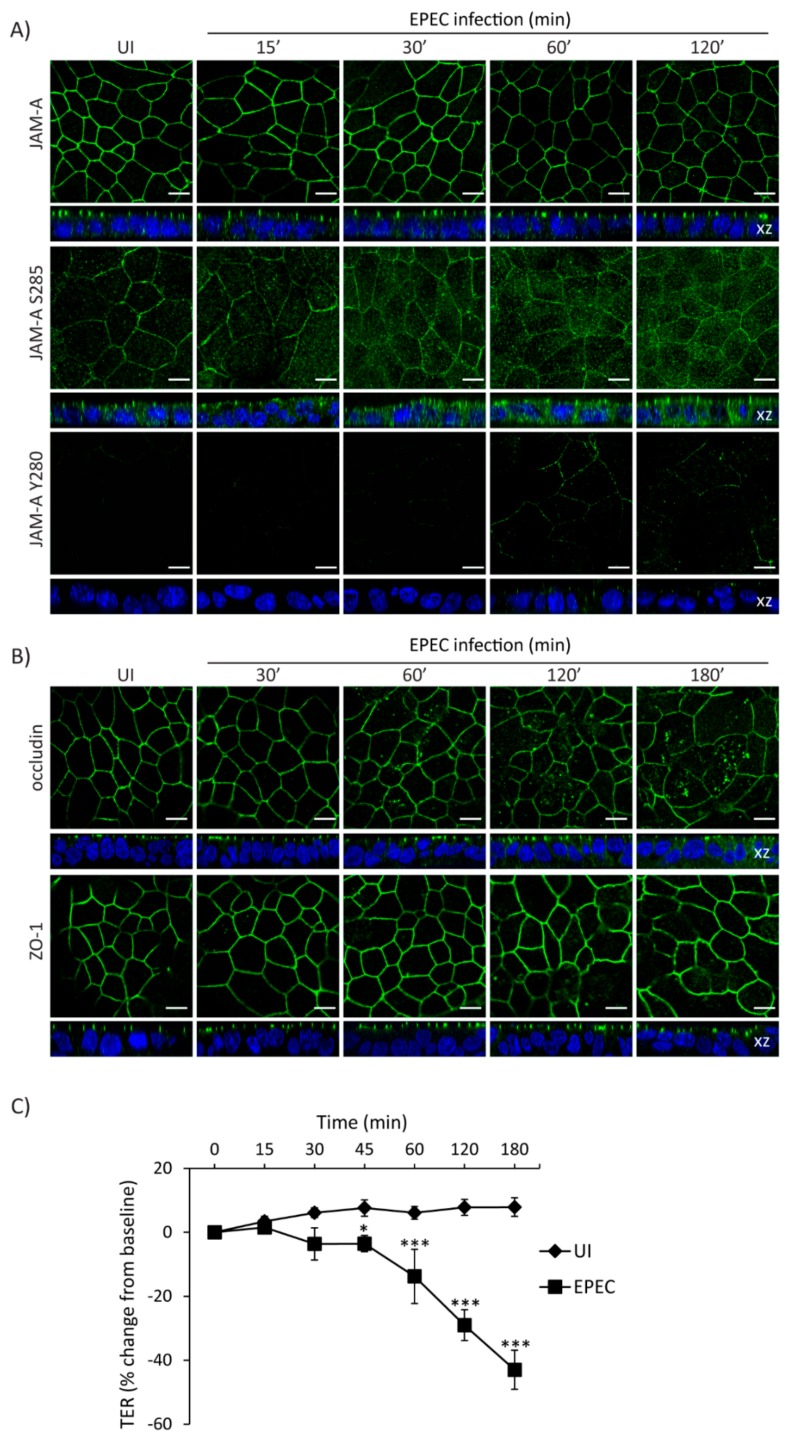
Temporal redistribution of TJ proteins and barrier dysfunction caused by EPEC. (**A–C**) SKCO-15 cells were plated on Transwells and infected or not (UI) with EPEC. Total JAM-A, JAM-A S285, JAM-A Y280, occludin, and ZO-1 localization and TER were determined. (**A**) EPEC does not alter the distribution of total JAM-A. In contrast, JAM-A S285 is displaced from the cell–cell contacts to the cytoplasm at 30 min post-infection. Tyrosine phosphorylation of JAM-A Y280 is apparent at 60–120 min post-infection. Scale bars, 10 µm. (**B**) EPEC induces the endocytosis of occludin and ZO-1 at 1 and 2 h post-infection, respectively. Scale bars, 10 µm. (**C**) TER drops significantly as early as 45 min post-infection and progressively decreases over time as more TJ proteins are displaced. TER reported as percent change from baseline. * *p <* 0.01, *** *p* < 0.001.

The interplay between cell polarity and TJ establishment and function is well defined. Active aPKCζ targets TJ proteins and participates in the assembly and maintenance of barrier function. EPEC impairs both cell polarity and intestinal epithelial TJ barrier function. To determine if the temporal sequence of TJ disruption following EPEC infection correlates with aPKCζ redistribution, SKCO-15 monolayers were infected with EPEC and the localization of TJ proteins and TER were analyzed over time. EPEC does not alter the localization of total JAM-A from the cell–cell contacts (Figure 5A). In contrast, JAM-A S285 is internalized by 30 min and TER significantly decreases 45 min post-infection (Figure 5A,C). Phosphorylation of JAM-A at tyrosine 280 (JAM-A Y280) is related to loss of barrier function [45]. Interestingly, we found that JAM-A Y280 is detectable only after 1–2 h EPEC infection, corresponding to leaky TJ (Figure 5A). Occludin disruption was seen at 1 h post-infection (Figure 5B) corresponding with a more significant drop in TER (–14.0 ± 8.0%, change from baseline) (Figure 5C). ZO-1 was the last TJ protein altered by EPEC moving into the cytoplasm at 2 h post-infection (Figure 5B) and corresponding to a profound drop in TER (–29.0 ± 5.0%, change from baseline) (Figure 5C).

**Figure 6:**
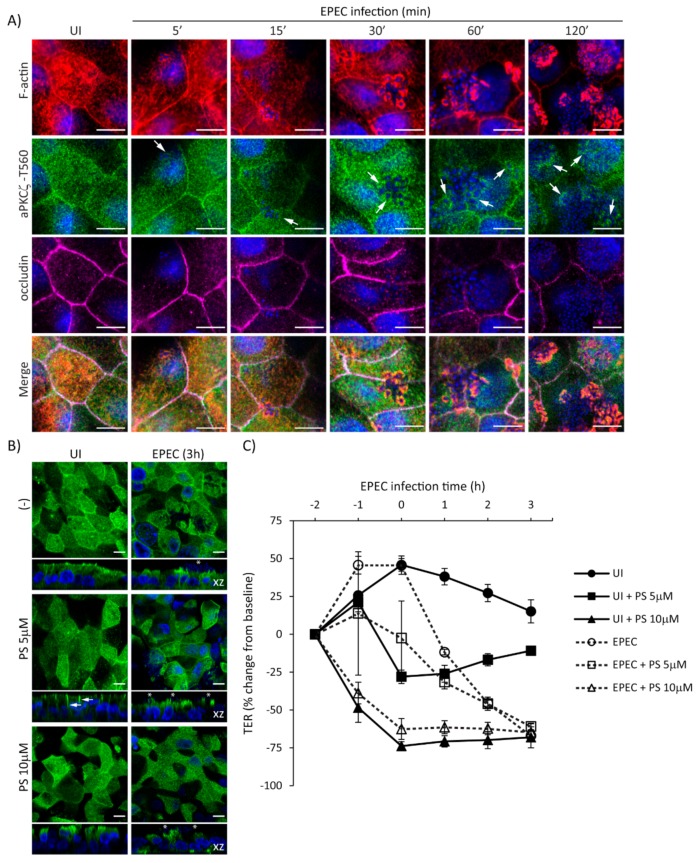
EPEC induces the recruitment of p-aPKCζ–T560 to actin pedestals prior to redistribution of occludin from cell borders. (**A**) SKCO-15 cells were plated on Transwells and infected or not (UI) with EPEC for 5–120 min. Immunofluorescence microscopy for F-actin, p-aPKCζ–T560, and occludin was performed. p-aPKCζ–T560 is recruited to pedestals co-localizing with actin consistently as early as 15 min and increases as infection progresses. Occludin localization is not displaced from cell borders until 1–2 h post-infection. Arrows indicate the presence of p-aPKCζ–T560 within EPEC pedestals. Scale bars, 10 µm. (**B** and **C**) SKCO-15 cells were plated on Transwells and pre-incubated or not with aPKCζ pseudosubstrate (PS) for 1 h prior to EPEC infection. Immunofluorescence of p-aPKCζ–T410 was performed and TER was measured. (**B**) Treatment with PS redistributes p-aPKCζ–T410 from the apical membrane and intercellular junctions to the lateral membrane (arrows) and cytoplasm without altering recruitment to EPEC pedestals (stars). Scale bars, 10 µm. (**C**) Inhibition of aPKCζ results in rapid disruption of barrier function in both UI and EPEC-infected monolayers.

To further delineate the temporal relationship of EPEC-induced recruitment of p-aPKCζ-T560 to pedestals and the redistribution of occludin from the cell–cell borders, localization of F-actin, p-aPKCζ-T560, and occludin, was assessed from 5–120 min post-infection. EPEC induces the recruitment of F-actin almost immediately upon bacterial attachment (5–15 min) (Figure 6A). Recruitment of p-aPKCζ–T560 to pedestals occurs as early as 5–15 min, and its co-localization with F-actin is seen by 15–30 min post-infection (Figure 6A). Consistent with our previous results, occludin remains at the cell borders at early time points after infection being displaced from the membrane at 60–120 min post-infection (Figure 6A). To examine if inhibition of aPKCζ activity affects its recruitment to EPEC pedestals and subsequent impairment in barrier function, SKCO-15 monolayers were incubated or not (-) with aPKCζ pseudosubstrate (PS) prior to EPEC infection, then localization of p-aPKCζ–T410 and TER were determined. Monolayers treated with PS alone show the redistribution of p-aPKCζ–T410 from the apical membrane and cell–cell contacts to the lateral membrane domain and cytoplasm, and cell death is apparent (Figure 6B). Interestingly, PS treatment does not alter the recruitment of p-aPKCζ–T410 to EPEC pedestals (Figure 6B). In agreement with previous reports [17], inhibition of aPKCζ with PS disrupts barrier function (–28.0% ± 4.0 and –73.0% ± 3.0 change from baseline, PS 5 µM and PS 10 µM, respectively) (Figure 6C). EPEC infection does not alter the drop in TER caused by PS (Figure 6C). Interestingly, monolayers incubated with PS alone (5µM) begin to recover TER (Figure 6C). In contrast, EPEC infection (2 h) in the presence of 5 µM PS causes a progressive drop in TER (–46.0% ± 4.0 change from baseline) similar to infection with EPEC alone (–45.0% ± 2.0 change from baseline) (Figure 6C), suggesting that the displacement of p-aPKCζ–T410 away from TJ results in a perturbation of barrier function. Together these results demonstrate the progressive dismantling of TJ by EPEC and the corresponding impact on barrier function, events that occur after the recruitment of active aPKCζ to actin pedestals.

## 3. Discussion

The present study provides evidence that EPEC perturbs PAR polarity complex integrity and induces the recruitment of aPKCζ to actin pedestals almost immediately following EPEC attachment. The SNX9-binding domain of EspF is important for the recruitment and organization of p-aPKCζ–T560 organization within EPEC pedestals, and the endocytosis of TJ proteins. We speculate that the very early recruitment of active aPKCζ to actin within pedestals and cell–cell contacts triggers downstream signalling events that ultimately disrupt intestinal epithelial TJ structure and function.

In view of the well-established interdependence between polarity and TJ complexes, we questioned if the apico-basal polarity defects caused by EPEC might be upstream of events that lead to TJ barrier perturbation. We provide evidence that EPEC perturbs PAR polarity complex integrity, mislocalizes active aPKCζ to the lateral membrane in vivo, and induces aPKCζ recruitment to pedestals and co-localization with actin at cell–cell borders almost immediately following EPEC attachment in vitro. These events precede the progressive dismantling of TJ proteins from the cell–cell contacts and barrier dysfunction. This is the first report correlating the temporal disruption of the PAR polarity complex with TJ disassembly by EPEC. Phosphorylated JAM-A S285, which is involved in TJ assembly [46], is displaced from cell–cell contacts at early times following EPEC infection. Subsequently, occludin then ZO-1 are displaced from TJ temporally correlating with the progressive loss of TER. Furthermore, pedestal formation and recruitment of active aPKCζ to pedestals occurs prior to occludin mislocalization. Interestingly, we found that EPEC induces the presence of phosphorylated JAM-A Y280 at intercellular contacts at times that correspond to barrier loss. Inflammatory stimuli increase JAM-A Y280 phosphorylation by balancing activity of the Src kinase, YES-1, and the phosphatase PTPN13, ultimately leading to disruption of barrier function [45]. EPEC induces a pro-inflammatory response through several signaling pathways resulting in the activation of NF-κB, ERK1/2, p38, JNK and PKCζ, and the upregulation of IL-8 expression, which contribute to intestinal barrier dysfunction [42,47,48,49]. EPEC also regulates the activity of multiple kinases, including the Src family, that contribute to actin polymerization [50,51]. Interestingly, Src family kinases contribute to Tir phosphorylation and actin pedestal formation [52,53]. Further analysis is required to understand how these pathways are involved in aPKCζ activity and relocalization. 

EspF and Map are major EPEC effectors that disrupt the Crb polarity complex and TJ, however, the mechanisms are not known. Here we demonstrate that EspF likely through binding SNX9 recruits active aPKCζ to actin within pedestals and at the plasma membrane. Map does not have a role in aPKCζ recruitment, but as aPKCζ also associates with actin in filopodia, we cannot discard the notion that Map may regulate aPKCζ activity during filopodia dynamics [33,54,55]. The EspF/SNX9/N-WASP complex participates in F-actin polymerization, membrane remodeling during EPEC pathogenesis, impairment of TJ structure and function, and recruitment of ZO-1 and ZO-2 to pedestals, but this complex does not affect EPEC pedestal formation [33,34,35,39,40]. This is in agreement with our data in which clear pedestals form after infection with *ΔespF*, however, the organization of total aPKCζ within pedestals and the recruitment of active aPKCζ to actin within pedestals and at cell–cell borders are severely altered following infection with *ΔespF* and *ΔespF/pespFD3*. Interestingly, EspF of rabbit EPEC (REPEC) and EPEC is involved in pedestal maturation [34,56], suggesting that EspF coordination of aPKCζ and actin within pedestals and at the membrane influences downstream signaling events that lead to TJ disruption.

EspF interacts with 14-3-3ζ [36], a protein that binds Par3 to regulate cell polarity [57]. This interaction is not involved in TJ barrier disruption by EPEC as determined by infection with *ΔespF/pSer47A* and *ΔespF/pSer47/50A* and is consistent with our finding that Par3 localization remains unchanged after EPEC infection. EspF also forms a complex with SNX9 and N-WASP. This binding is required to disrupt Crb3, ZO-1, and E-cadherin from cell–cell contacts increasing their cytoplasmic accumulation, thus leading to impaired cell polarity and barrier function [32,40]. We found that EspF is essential for the displacement of JAM-A S285 and occludin from cell–cell contacts, as well as disruption of barrier function in SKCO-15 cells, as occurs in Caco-2 and T84 cells [33,40]. While the EspF–SNX9 interaction is important for barrier disruption in SKCO-15 and Caco-2, it does not play a role in T84 cells, as infection with the EspF-D3 mutant failed to protect against a loss of TER and the redistribution of occludin in polarized T84 cells [33]. Furthermore, examination of pedestals in EPEC-infected T84 cells reveals that aPKCζ and actin co-localization is not impacted by the EspF-D3 mutant. PKC activity post-EPEC-infection also differs between T84 and SKCO-15 cells [41,42]. Although all derived from colonic cancer cell lines, Caco-2 when grown for extended periods differentiate into small intestine-like cells, whereas SKCO-15 and T84 cells are more colonic in nature. Several studies have focused on the biochemical and structural differences between these colonic cell lines [58,59,60]. In addition, SNX9 expression levels differ between colon cancer cell lines, as well as having other varying redundant sorting nexin proteins [61,62,63]. These data highlight the cell-specific aPKCζ responses and different mechanisms that contribute to TJ barrier dysfunction and lends support to the notion that mislocalization of aPKCζ activity contributes to TJ disruption in SKCO-15 cells. 

EPEC modulates clathrin-mediated endocytosis, thus it has been suggested that EPEC-induced TJ disassembly may occur via this mechanism. For instance, EspF recruits clathrin, AP2, early (Rab5a and EEA1) and recycling (Rab4a, Rab11a, Rab11b, FIP2, Myo5b) endocytic proteins to sites of infection [38]. The EspF binding partner SNX9 is recruited to clathrin-coated pits and associates with N-WASP, dynamin, Arp2/3 and other associated proteins to promote endocytosis of plasma membrane receptors [64,65,66,67,68,69]. In addition, EPEC mediates Crb3 endocytosis in a dynamin-dependent manner [32]. However, data presented herein indicate that EPEC-induced endocytosis of occludin and disruption of barrier function occur via a dynamin-independent mechanism indicating that an alternative endocytic pathway is responsible and supports aPKCζ involvement in TJ disassembly.

Interestingly, aPKCζ has direct and indirect roles in the formation and maintenance of polarity and TJ structure and function. aPKCζ phosphorylates the TJ proteins occludin, JAM-A, claudin-4, and ZO-1 to establish and maintain TJ structure and barrier function [17,46,70,71]. Silencing of aPKCζ or inhibition of its kinase activity with PS are associated with dephosphorylation of occludin and ZO-1, delayed assembly and perturbed maintenance of barrier function [17]. We found that PS treatment mislocalizes p-aPKCζ–T410 to the lateral membrane and cytoplasm but does not affect its recruitment to EPEC pedestals. In addition, when aPKCζ is absent from the lateral membrane, whether due to high concentrations of PS or recruitment to EPEC pedestals, barrier function is severely altered. aPKCζ also phosphorylates polarity proteins including Crb3, Par3, Lgl, and Par1b, and is dependent on interactions with Par6 and Cdc42 for its activity at the apical domain [13,72,73,74,75]. aPKC activation during endothelial morphogenesis is determined by the adaptor protein Nck [76]. After Nck is recruited to actin pedestals [77,78], it could serve as a hub for aPKCζ pedestal activity. Therefore, one could speculate that the early recruitment of aPKCζ to pedestals redirects its kinase activity away from TJ and polarity complexes, thus disrupting apico-basal polarity and barrier function.

Besides phosphorylation of polarity and TJ proteins, aPKCζ plays a role in actin dynamics. In *Drosophila*, Cdc42 and active aPKC cause the interaction of DSH3PX1/Dock/Wasp/Arp2/3, homologues of human SNX9/Nck/N-WASP/Arp2/3. This complex is involved in actin rearrangement and adherens junction stability [79,80,81]. Interestingly, we found that the EspF–SNX9 interaction was essential not only for the highly organized structure of aPKCζ and actin within pedestals but also for increased aPKCζ and actin co-localization at the membrane. aPKC activates ezrin, a plasma membrane protein that links and stabilizes the actin cytoskeleton to the membrane facilitating microvilli formation and endocytosis [82,83,84]. Ezrin and SNX9 are enriched on curved membranes; ezrin may interact with SNX9 via its curvature-sensing I-BAR domain facilitating ezrin to tether and close membranes during membrane tubule formation [62,85]. EspF/SNX9 binding is involved in F-actin polymerization and promotes the formation of tubular vesicles in EPEC-infected cells [33,35,39]. We previously determined that ezrin recruitment to the cytoskeleton is dependent on EspF, and ezrin activation contributes to disruption of TJ barrier function [86]. EspF, through its interaction with several host proteins including actin, profilin, Arp2, N-WASP, Abcf2, cytokeratin 18, WIPF1, SNX9/18/33, regulates cytoskeletal dynamics [33,34,36,37,38]. Together, these data indicate that through EspF interactions with actin-binding proteins and its structured localization of aPKCζ with actin at the membrane, aPKCζ activity likely participates in actin dynamics controlling endocytic pathways and TJ maintenance following EPEC infection. 

**Figure 7:**
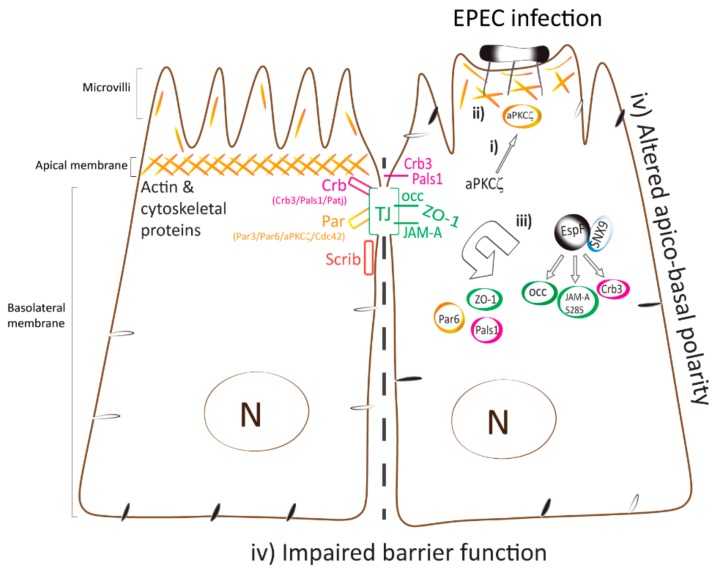
Model displaying the temporal mislocalization of aPKCζ leading to the dismantling of polarity and TJ complexes. Polarized epithelial cells consist of the apical membrane facing the lumen and the basolateral domain in contact with the underlying basement membrane. TJ, localized at the most apical area of the lateral membrane, contribute to the maintenance of apico-basal polarity. Polarity complexes (Crb, PAR, and Scrib) localize along the lateral membrane and modulate TJ assembly and function. (**i**) EPEC infection induces the recruitment of active aPKCζ to actin pedestals and at plasma membrane almost immediately upon bacterial attachment. (**ii**) Actin and aPKCζ are highly organized within EPEC pedestals via a mechanism that is dependent on the EspF/SNX9 binding domain. (**iii**) TJ (JAM-A S285, occludin, and ZO-1) and polarity (Par6, Crb3, and Pals1) proteins are displaced from cell–cell contacts and internalized into the cytoplasm. (**iv**) Apico-basal polarity, evidenced by redistribution of basolateral proteins to the apical membrane [32], and TJ are perturbed. These events are largely dependent on EspF–SNX9 binding domain, although other effectors may contribute.

The recruitment of cytoskeletal, adaptor, and signaling proteins into pedestals, as well as rearrangement of the actin cytoskeleton in host cells during EPEC infection are crucial steps in EPEC pathogenesis [77,87,88,89,90]. Together the results reported herein suggest a mechanism by which EPEC perturbs intestinal epithelial TJ (Figure 7). EPEC recruits active aPKCζ to interact with actin within pedestals and at the membrane of cell–cell contacts immediately following bacterial attachment. The recruitment of active aPKCζ away from polarity complexes and into highly organized actin pedestals via an EspF–SNX9 dependent process may be the initial upstream event that triggers downstream pathways that alter actin dynamics and disrupt intestinal epithelial TJ structure and function thus contributing to EPEC pathogenesis.

## 4. Materials and Methods

### 4.1. Tissue Culture

SKCO-15 cell line derived from human adenocarcinoma of the colon displays typical intestinal epithelial cell features with adhesion complexes and microvilli [59,60]. SKCO-15 cultures were grown in Dulbecco’s Modified Eagle Medium (DMEM) complemented with 10% fetal bovine serum with antibiotics as previously described [32]. T84 cells were grown in low glucose DMEM/Ham’s F12 (Gibco) medium complemented with 10% newborn calf serum (Gibco, Life Technologies, Carlsbad, CA, USA) and antibiotics as previously reported [91]. Cells were grown at 37 °C in 5% CO2, once monolayers were confluent (7–10 days), media was replaced with bacterial growth medium 24 h prior to infection.

### 4.2. Antibodies and Reagents

Par3 (07-330, EMD Millipore), Par6 (ab49776 and ab6022, Abcam, Cambridge, MA, USA), aPKCζ (sc-17781, Santa Cruz Biotechnology, Dallas, TX, USA), p-aPKCζ–T560 (ab62372, Abcam, Cambridge, MA, USA), p-aPKCζ–T410 (sc-12894R, Santa Cruz Biotechnology, Dallas, TX, USA), actin (A2066, Sigma-Aldrich, St. Louis, MO, USA), F-actin BODIPY 558/568 Phalloidin (B3475, Invitrogen, Life Technologies Carlsbad, CA, USA), occludin (33-1500, Invitrogen, Life Technologies, Carlsbad, CA, USA), JAM-A S285 (sc-17430, Santa Cruz Biotechnology, Dallas, TX, USA), JAM-A Y280 (600-401-GN5, Rockland Immunochemicals Inc, Limerick, PA), JAM-A (361700, Invitrogen, Life Technologies, Carlsbad, CA, USA), and ZO-1 (61-7300, Invitrogen, Life Technologies, Carlsbad, CA, USA). Secondary antibodies used for immunofluorescence were Alexa Fluor (Life Technologies, Carlsbad, CA, USA). Dynasore (Sigma-Aldrich, St. Louis, MO, USA) was used at 80 µM. aPKCζ pseudosubstrate (539624, Millipore-Sigma, Burlington, MA, USA) was used at 5 and 10 µM. 

### 4.3. Bacterial Culture

The following EPEC strains were used: wt EPEC 0127:H6 E2348/69, *ΔespF* [92], *ΔespF*/p*espF*, *Δmap* [93], *ΔespF/pespF*D3 [33], *ΔespF/pSer47A* and *ΔespF/pSer47/50A* [Hecht unpublished]. Bacterial cultures were grown overnight in Luria-Bertani broth with appropriate selective antibiotics. For infections, bacterial cultures were processed as previously reported [32,91]. Monolayers plated on Transwells (Costar #3740), or six-well plates were infected with bacterial strains at a multiplicity of infection of 50. Infected cells were incubated at 37 °C in 5% CO2 for indicated times.

### 4.4. Murine Infection

Male mice C57BL/6J from 8 to 10 weeks old were used (Jackson Laboratory, Bar Harbor, ME, USA), and housed in a specific pathogen-free facility at Loyola University Chicago (LUC) Medical Campus. LUC Animal Care and Use Committee approved all animal protocols. Animals were infected with EPEC by oral gavage and sacrificed on day three post-infection [94]. Intestinal tissues were processed for immunofluorescence as reported [91]. 

### 4.5. Immunofluorescence

Cells were plated on coverslips or Transwells and fixed with cold methanol at –20 °C for 10 min or with 4% PFA for 15–30 min and permeabilized with 0.1% Triton X-100 (5–15 min). Samples were blocked and incubated with primary antibodies overnight at 4 °C with Invitrogen blocking solution, washed with PBS, then processed for immunofluorescence. Paraffin embedded intestinal sections of infected mice were processed as previously described [91]. 

### 4.6. Imaging

Slides were analyzed using a confocal Leica TCS SPE DMI 4000B (LAS X software, Leica, Wetzlar, Germany) microscope. Z-stack images were acquired in 0.33 µm sections and processed using the 3-D volume setting of LasX software. Images were processed using Adobe Photoshop CC 2018 and FIJI-ImageJ-64 software. Heat maps were generated from red and green channels using ImageJ software (Analyze> Colocalization> colocalization threshold > show colocalized pixel map), then channels were split and co-localization channel assigned LUT > Fire and auto-contrasted. Quantitation of p-PKCζ–T560 and F-actin were accomplished with ImageJ. Green and red channels were analyzed separately. A 35-pixel length line was drawn and centered over cell–cell contacts or pedestals, and the line duplicated in each channel (Edit > selection > restore selection). Pixel intensity was measured over the length of the line (Analyze > plot profile), recorded for at least 15 regions of interest from membranes and pedestals of three biological replicates and statistical analysis was performed as described below.

### 4.7. Western Blot Analysis

SKCO-15 monolayers were rinsed twice with cold PBS, protein extraction was achieved with RIPA buffer containing protease inhibitors as previously reported [32]. Cell lysates were processed by electrophoresis (SDS-PAGE), transferred to Immobilon membrane (IPFL00010, Millipore, Burlington, MA, USA). Membranes were incubated with primary antibodies overnight at 4 °C and LI-COR secondary antibodies for 1 h at room temperature. Immunoblotting was performed using LI-COR Odyssey system (LI-COR Bioscience, Lincoln, NE, USA)

### 4.8. Generation of Tet-On System

SKCO-15 cells were co-transfected with p-RetroX–Tet–On Advanced and pRetroX–Tight–Pur–EspF–HA [32]. To generate stable cell lines, transfected SKCO-15 cells were selected with G418 and puromycin for 2–3 weeks. Expression of the transgene pRetroX–Tight–Pur–EspF–HA was induced in presence of doxycycline (500 ng/mL) for three days [32]. 

### 4.9. Measurement of Transepithelial Electrical Resistance

Wild type and Tet–On SKCO-15 cells (3 × 10^5^ cells) were plated in triplicate on Transwell filters in DMEM. TER was measured using cellZscope (nanoAnalytics, Munster, Germany) for 5–7 days. Cells were infected with wt EPEC or mutant strains and TER measurements were taken at indicated intervals. Expression of pRetroX–Tight–Pur–EspF–HA was induced with doxycycline (+dox) and measurements were followed for 3–6 days. TER values were normalized to UI monolayers.

### 4.10. PKC activity Assays

SKCO-15 and T84 cells were grown in 6-well plates and infected for the indicated time points. PKC Kinase Activity Assay Kit (ab139437, Abcam, Cambridge, MA) was used according to the manufacturer’s instructions.

### 4.11. Statistical Analysis

All experiments were performed in triplicate. TER results are the mean ± SEM of three independent experiments performed in triplicate. *P* values were calculated by ANOVA Tukey’s Multiple Comparison Test and Pearson correlation co-efficient using GraphPad Prism v7. 

## Supplementary Materials

Supplementary materials can be found at https://www.mdpi.com/1422-0067/21/2/527/s1.

## Abbreviations

aPKCζ	Atypical protein kinase C zeta
EPEC	Enteropathogenic *Escherichia coli*
F-actin	Filamentous actin
JAM-A S285	Junctional adhesion molecule-A phosphorylated at Serine 285
JAM-A Y280	Junctional adhesion molecule-A phosphorylated at Tyrosine 280
p-aPKCζ-T410	aPKCζ phosphorylated at Threonine 410
p-aPKCζ-T560	aPKCζ phosphorylated at Threonine 560
PS	aPKCζ pseudosubstrate inhibitor
SNX9	Sorting nexin 9
TJ	Tight junctions
TER	Transepithelial electrical resistance
ZO-1	Zonula Occludens -1
