# Supplementary material for: Enteropathogenic Escherichia coli (EPEC) Recruitment of PAR Polarity Protein Atypical PKCζ to Pedestals and Cell–Cell Contacts Precedes Disruption of Tight Junctions in Intestinal Epithelial Cells
Source: Int J Mol Sci. 2020 Jan 14;21(2):527. doi: 10.3390/ijms21020527 (PMC7014222; doi:10.3390/ijms21020527)
Supplement: Supplementary file 1 [file ijms-21-00527-s001.pdf]

International Journal of
Molecular Sciences

MDPI

Supplemental material

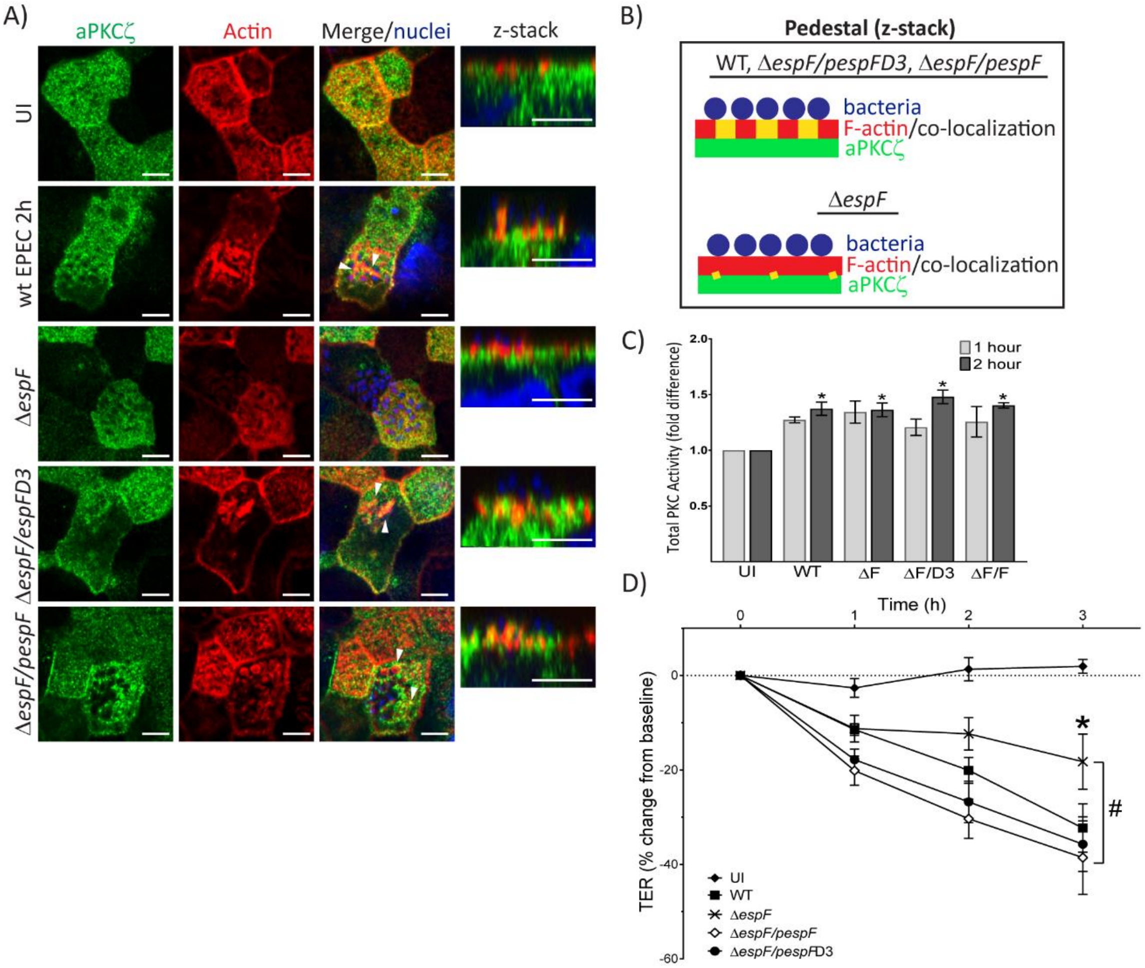

Supplemental Figure 1. EspF/SNX9 binding is dispensable in T84 cells for aPKCζ and F-actin pedestal organization, PKC activity, and TJ disruption. T84 monolayers were infected, or not (UI), with wt EPEC, *ΔespF*, or *ΔespF* complemented with mutated *espF* (*ΔespF*/*pespF*D3) or wt *espF* (*ΔespF*/*pespF*) to assess the localization of aPKCζ and F-actin, PKC kinase activity and TER. (A) aPKCζ aggregates and co-localizes with F-actin under attached bacteria 2 hours post-infection with wt EPEC, *ΔespF*/*pespF*D3 or *ΔespF*/*pespF*. In contrast, reduced co-localization is apparent after infection with *ΔespF*. Arrowheads indicate regions of co-localization between aPKCζ and F-actin. Scale bars: 10µm (en face); 5µm (z-stack). (B) Schematic representation of aPKCζ (green), F-actin (red), and co-localization (yellow) within pedestals following infection with wt EPEC and EspF mutant strains. (C) Significant increase in PKC activity 2 hours post-infection with wt EPEC and EspF mutant strains compared to UI monolayers. *p<0.05. (D) All EPEC strains significantly reduced TER 3 hours post-infection compared to UI #p<0.001. TER is significantly higher after infection with *ΔespF*, but not *ΔespF*/*pespF*D3, compared to wt EPEC infection *p<0.05. TER reported as percent change from baseline.
